# Extract of *Nicotiana tabacum* as a potential control agent of *Grapholita molesta* (Lepidoptera: Tortricidae)

**DOI:** 10.1371/journal.pone.0198302

**Published:** 2018-08-23

**Authors:** Souvic Sarker, Un Taek Lim

**Affiliations:** 1 Department of Plant Medicals, Andong National University, Andong, Republic of Korea; 2 Institute of Agricultural Science and Technology, Andong National University, Andong, Republic of Korea; Ecole des Mines d'Ales, FRANCE

## Abstract

Oriental fruit moth, *Grapholita molesta* (Busck) (Lepidoptera: Tortricidae), is an important pest of stone and pome fruits. Growers usually depend on chemical insecticides to control this pest, but demand for more environmentally-friendly means of controlling pests is increasing. At least 91 plant extracts have been reported to be effective against other lepidopterans, but their acute toxicity against *G*. *molesta* has rarely been studied. Among these 91 materials, we assessed the residual toxicity of 32 extracts against first instar larvae (< 5 h old) of *G*. *molesta* in the laboratory. *Nicotiana tabacum* L., used at the concentration of 2 mg/ml, showed the highest corrected mortality (92.0%) with a lethal time (LT_50_) value of 12.9 h. The extract was followed in its efficacy by *Allium sativum* L. (88.0%), *Zanthoxylum piperitum* (L.) De Candolle (70.0%), and *Sapindus mukorossi* Gaertner (65.0%), when mortality was assessed at 20 h after exposure. Against adult fruit moths (< 5 d old), *N*. *tabacum* also showed the highest corrected mortality among tested extracts, being 85 and 100% in adult females and males, respectively, at 168 h after exposure. However, there was no synergistic effect of the combined application of any of the top four extracts in either laboratory or greenhouse assays. Oviposition by *G*. *molesta* on peach twigs was reduced 85–90% when *N*. *tabacum* was applied at 4 ml/ twig compared to control (methanol), demonstrating that *N*. *tabacum* may have potential for use as a botanical insecticide against *G*. *molesta*.

## Introduction

Oriental fruit moth, *Grapholita molesta* (Busck) (Lepidoptera: Tortricidae), is a serious pest of fruit trees in the temperate regions, worldwide [1–4]. Its host range encompasses species within the family Rosaceae, mostly those from the genera *Prunus* and *Pyrus* [1]. Stone fruit peach [*Prunus persica* L. (Rosales: Rosaceae)] is considered the primary host of *G*. *molesta* whereas the pome fruits pear [*Pyrus communis* L. (Rosales: Rosaceae)] and apple [*Malus domestica* L. (Rosales: Rosaceae)] are considered secondary hosts [5].

Application of organophosphorus, carbamates, or synthetic pyrethroid pesticides is a common method for control of *G*. *molesta* in Korea [6, 7], but the development of insecticide resistance is a serious threat to the fruit industry [6], and *G*. *molesta* has developed resistance to 14 insecticides including 10 organophosphates [8]. As many of these insecticides are neurotoxins, they have some potential to be harmful to non-target organisms, including people and domestic animals [4]. To avoid such risks, new pest management tactics need to be developed for the management of *G*. *molesta*. Due to their less residual toxicity, lower development cost, and general safety to people, plant extracts have the potential to be effective alternatives for control of pest insects [9].

Secondary plant metabolites, such as polyphenols, terpenoids, alkaloids, steroids, lignans, essential oils, fatty acids, and sugars, are regarded as defense mechanisms against insect attack [10]. Some secondary metabolites inhibit insect development and reproduction, while others act as antifeedants, repellents, or fumigants [11–13]. Botanical insecticides degrade quickly, meaning their impact on beneficial or non-target organisms is less than that of conventional insecticides [14], thus would be more compatible with biological control agents than synthetic insecticides. Furthermore, botanical insecticides have also multiple modes of action, development of resistance in insects has been reported less frequently [15].

At least 91 plant extracts have been found effective against pest lepidopterans in studies published from 2000–2015 (Table 1). Some of these extracts have demonstrated a similar level of pest toxicity as synthetic insecticides. Extracts from goat weed (*Ageratum conyzoides* L.) and siam seed (*Chromolaena odorata* [L.]) controlled *Plutella xylostella* L. larvae, a rate similar to the synthetic insecticide emamectin benzoate [16]. Antifeedant activity was found for extracts of *Chrysanthemum* sp. and *Achillea millefolium* L. against *Spodoptera littoralis* (Boisduval) and *Pieris rapae* L., respectively [17, 18], and plant extracts have also been found to act as an oviposition deterrent; Reegan et al. [19] reported that a hexane extract of *Limonia acidissima* (L.) showed 100% oviposition deterrency for adults females of *Culex quinquefasciatus* Say and *Aedes aegypti* L.

**Table 1:** Plant extracts reported during 2000–2015 to show toxicity against lepidopteran insects.

Plant species	Plant parts	Solvent	Lepidopteran insects tested	
			Species	Family
*Abrus precatorius* [38]	Seed	Ethanol	*Galleria mellonella*	Pyralidae
*Achillea millefolium* [18]	Leaf	Methanol	*Pieris rapae*	Pieridae
*Acorus calamus* [39]	Rhizome	Ether	*Sitotroga cerealella*	Gelechiidae
*Ageratum conyzoides* [16]	Leaf	Detergent	*Plutella xylostella*	Yponomeutidae
*Allium cepa* [40]	Fresh onion	Tween 20	*Tuta absoluta*	Gelechiidae
*Allium sativum* [40]	Fresh garlic	Tween 20	*Tuta absoluta*	Gelechiidae
*Alpinia galanga* [41]	Rhizome	Ethanol	*Plutella xylostella*	Yponomeutidae
*Anona coriacea* [42]	Leaf	Methanol	*Spodoptera frugiperda*	Noctuidae
*Anona dioica* [42]	Leaf	Methanol	*Spodoptera frugiperda*	Noctuidae
*Anona muricata* [43]	Leaf	Ethanol	*Plutella xylostella*	Yponomeutidae
*Artemisia annua* [18]	Leaf	Methanol	*Pieris rapae*	Pieridae
*Artemisia vulgaris* [44]	Whole plant	Methanol	*Spodoptera littoralis*	Noctuidae
*Avicennia marina* [45]	Aerial part	Hexane	*Phthorimaea operculella*	Gelechiidae
*Azadirachta indica* [46]	Seed	Water	*Tuta absoluta*	Gelechiidae
*Bifora radiens* [47]	Whole plant	Acetone	*Thaumetopoea solitaria*	Thaumetopoeidae
*Cabralea canjerana* [48]	Seed/ Fruit	Ethanol	*Spodoptera frugiperda*	Noctuidae
*Capparis aegyptia* [45]	Aerial part	Hexane	*Phthorimaea operculella*	Gelechiidae
*Capsicum annum* [49]	Leaf	Methyl. chloride	*Spodoptera littoralis*	Noctuidae
*Capsicum frutescens* [16]	Fruit	Detergent	*Plutella xylostella*	Yponomeutidae
*Carica papaya* [50]	Seed	Methanol	*Spodoptera frugiperda*	Noctuidae
*Cassia sophera* [16]	Leaf	Detergent	*Plutella xylostella*	Yponomeutidae
*Chromolaena chaseae* [51]	Leaf	Ethanol	*Spodoptera frugiperda*	Noctuidae
*Chromolaena odorata* [16]	Leaf	Detergent	*Plutella xylostella*	Yponomeutidae
*Chrysanthemum grandiflorum* [17]	Aerial part	Metanol	*Spodoptera littoralis*	Noctuidae
*Chrysanthemum indicum* [52]	Leaf	Water	*Plecoptera reflexa*	Noctuidae
*Chrysanthemum macrotum* [17]	Aerial part	Methanol	*Spodoptera littoralis*	Noctuidae
*Chrysanthemum morifolium* [53]	Leaf	Methanol	*Trichoplusia ni*	Noctuidae
*Chrysanthemum segetum* [17]	Aerial part	Methanol	*Spodoptera littoralis*	Noctuidae
*Citrullus colosynthis* [54]	Seed	Ammonium sulfate	*Ectomyelois ceratoniae*	Pyralidae
*Citrus sinensis* [55]	Leaf	Phenol	*Phyllocnistis citrella*	Gracillariidae
*Cleome deoserifolia* [44]	Aerial part	Ethanol	*Phthorimaea operculella*	Gelechiidae
*Cleome spinosa* [56]	leaves	Ethanol	*Pieris rapae*	Pieridae
*Commiphora molmol* [57]	Stem	Water	*Spodoptera littoralis*	Noctuidae
*Croton urucurana* [58]	Stem	Methanol	*Anagasta kuehniella*	Pyralidae
*Cymbopogon martinii* [59]	Whole part	Water	*Euprosterna elaeasa*	Limacodidae
*Cyprus rotundus* [41]	Tuber	Ethanol	*Plutella xylostella*	Yponomeutidae
*Datura metel* [60]	Leaf	Methanol	*Helicoverpa armigera*	Noctuidae
*Delphinium consolida* [44]	Whole plant	Methanol	*Spodoptera littoralis*	Noctuidae
*Dimorphandra mollis* [61]	Leaf	Ethanol	*Sitotroga cerealella*	Gelechiidae
*Euphorbia lathyrus* [62]	Seed	Ethanol	*Spodoptera littoralis*	Noctuidae
*Fumaria officinalis* [47]	Whole plant	Acetone	*Thaumetopoea solitaria*	Thaumetopoeidae
*Ginkgo biloba* [63]	Seed coat	Methanol	*Spodoptera exigua*	Noctuidae
*Glycine max* [64]	Leaf	Isooctane	*Heliothis zea*	Noctuidae
*Gomphrena globosa* [41]	Seed	Ethanol	*Plutella xylostella*	Yponomeutidae
*Hordium sativum* [38]	Seed	Ethanol	*Galleria mellonella*	Pyralidae
*Hovenia dulcis* [65]	Leaf	Water	*Anticarsia gemmatalis*	Erebidae
*Humulus lupulus* [47]	Whole plant	Methanol	*Thaumetopoea solitaria*	Thaumetopoeidae
*Hymenoxys robusta* [66]	Leaf	Methanol	*Spodoptera exigua*	Noctuidae
*Ipomoea pauciflora* [67]	Seed	Hexane	*Spodoptera frugiperda*	Noctuidae
*Jatropha curcas* [16]	Leaf	Detergent	*Plutella xylostella*	Yponomeutidae
*Jatropha gossypifolia* [68]	Leaf	Ethanol	*Spodoptera frugiperda*	Noctuidae
*Laurus nobilis* [38]	Seed	Ethanol	*Galleria mellonella*	Pyralidae
*Lepidaploa lilacina* [51]	Leaf	Ethanol	*Spodoptera frugiperda*	Noctuidae
*Lychnophora ericoides* [51]	Leaf	Ethanol	*Spodoptera frugiperda*	Noctuidae
*Lychnophora ramosissima* [51]	Leaf	Ethanol	*Spodoptera frugiperda*	Noctuidae
*Melia azedarach* [68]	Leaf	Ethanol	*Spodoptera frugiperda*	Noctuidae
*Millettia ferruginea* [69]	Seed	Water	*Busseola fusca*	Noctuidae
*Momordica charantia* [70]	Leaf	Methanol	*Leucoptera coffeella*	Lyonetiidae
*Nerium indicum* [71]	Seed	Water	*Helicoverpa assulta*	Noctuidae
*Nicotiana tabacum* [16]	Leaf	Detergent	*Plutella xylostella*	Yponomeutidae
*Ocimum gratissimum* [16]	Leaf	Detergent	*Plutella xylostella*	Yponomeutidae
*Pachyrhizus erosus* [72]	Seed	Methanol	*Plutella xylostella*	Yponomeutidae
*Peganum harmala* [73]	Leaf	Methanol	*Spodoptera exigua*	Noctuidae
*Pelargonium zonale* [40]	Leaf	Tween 20	*Tuta absoluta*	Gelechiidae
*Petroselium sativum* [38]	Seed	Ethanol	*Galleria mellonella*	Pyralidae
*Peumus boldus* [74]	Leaf	Water	*Spodoptera frugiperda*	Noctuidae
*Piper amalago* [75]	Leaf	Ethanol	*Tuta absoluta*	Gelechiidae
*Piper glabratum* [75]	Leaf	Ethanol	*Tuta absoluta*	Gelechiidae
*Piper mikanianum* [75]	Leaf	Ethanol	*Tuta absoluta*	Gelechiidae
*Plantago lanceolata* [70]	Leaf	Methanol	*Leucoptera coffeella*	Lyonetiidae
*Plantago psyllium* [38]	Seed	Ethanol	*Galleria mellonella*	Pyralidae
*Pongamia pinnata* [76]	Seed	Chloroform	*Earias Vittella*	Noctuidae
*Psychotria goyazensis* [77]	Leaf	Ethanol	*Spodoptera frugiperda*	Noctuidae
*Psychotria prunifolia* [61]	Leaf	Ethanol	*Sitotroga cerealella*	Gelechiidae
*Quassia amara* [78]	Wood	Methanol	*Hypsipyla grandella*	Pyralidae
*Ricinus communis* [79]	Leaf	Hexane	*Spodoptera frugiperda*	Noctuidae
*Rhododendron molle* [80]	Flower	Ethyl acetate	*Hypsipyla grandella*	Pyralidae
*Ruta chalepensis* [81]	Leaf	Hexane	*Hypsipyla grandella*	Pyralidae
*Sapindus mukorossi* [82]	Fruit	Water	*Thysanoplusia orichalcea*	Noctuidae
*Siphoneugena densiflora* [83]	Leaf	Methanol	*Spodoptera frugiperda*	Noctuidae
*Synedrella nodiflora* [19]	Leaf	Detergent	*Plutella xylostella*	Yponomeutidae
*Tagetes erecta* [84]	Leaf	Ethanol	*Spodoptera frugiperda*	Noctuidae
*Tanacetum mucroniferum* [44]	Whole plant	Methanol	*Spodoptera littoralis*	Noctuidae
*Tanacetum zahlbruckneri* [85]	Flower	Methanol	*Spodoptera littoralis*	Noctuidae
*Tithonia diversifolia* [61]	Leaf	Ethanol	*Sitotroga cerealella*	Gelechiidae
*Trichilia pallens* [86]	Twig	Water	*Spodoptera frugiperda*	Noctuidae
*Trichilia pallida* [86]	Twig	Water	*Spodoptera frugiperda*	Noctuidae
*Trichogonia villosa* [51]	Leaf	Ethanol	*Spodoptera frugiperda*	Noctuidae
*Vernonia holosenicea* [51]	Leaf	Ethanol	*Spodoptera frugiperda*	Noctuidae
*Zanthoxylum limonella* [87]	Bark	Ethyl acetate	*Spodoptera litrura*	Noctuidae
*Zea diploperennis* [88]	Leaf	Water	*Spodoptera frugiperda*	Noctuidae

As botanical insecticides are a potential alternative to conventional insecticides [9], the present study was conducted to assess the efficacy of various plant extracts against *G*. *molesta*. Among the 91 plant extracts reported in the literature, we could obtain only 32 plant extracts available and measured their acute toxicities against first instar larva and adults of *G*. *molesta*. We also evaluated the deterrent effect of these plant extracts on the oviposition of *G*. *molesta* females in the laboratory and under semi-field condition.

## Materials and methods

### Insect rearing procedures

Apples infested with oriental fruit moth were collected and kept in ventilated plastic containers (24.0 L × 17.0 W × 8.0 H cm) at 24.9 ± 0.1°C, 50.2 ± 1.3% RH, and a 16:8 h (L:D) photoperiod in an incubator (DS-11BPL, Dasol Scientific Co. Ltd, Hwaseong, Republic of Korea). When the larvae reached the fifth instar, they emerged from the apple and built their cocoons in the paper towel provided for pupation. Pupae were collected and held in breeding dishes (10.0 D × 4.0 H cm, 310102, SPL, Pocheon, Republic of Korea). When adult moths emerged, they were transferred into ventilated acrylic cylinders (25.5 H × 8.5 D cm), and provided with a piece of cotton soaked in 10% sugar solution as a food source. The acrylic cylinders were kept in a desiccator (36.0 L × 28.0 W × 25.0 H cm) and incubated at 25.6 ± 0.1°C and 91.2 ± 0.1% RH. When moths started to lay eggs on the wall, the cylinder was changed daily to collect freshly laid eggs. Acrylic cylinders bearing eggs on the walls were kept in a separate incubator at 25.6 ± 0.1°C and 91.2 ± 0.1% RH until egg hatch, after which first instar larvae were collected for the experiments or reuse in mass rearing.

### Extract preparation

Methanol extracts of test plants were purchased from KPEB (Korea Plant Extract Bank, Cheongju, Republic of Korea) (Table 2). Extraction consisted of extraction, filtering and yield testing, concentration, drying, and storage (http://extract.kribb.re.kr).

**Table 2:** Thirty-two plant extracts evaluated in this study.

Plants (Reference number)	Extracted part	Family name	Plants (Reference number)	Extracted part	Family name
*Gomphrena globosa* L. (036–080)	Whole plant	Amaranthaceae	*Ginkgo biloba* L. (031–069)	Leaf-stem	Ginkgoaceae
*Allium cepa* L. (034-064)	Whole plant	Amaryllidaceae	*Piper Kadzura* Ohwi (001–223)	Leaf	Piperaceae
*Allium sativum* L. (033–033)	Whole plant	Amaryllidaceae	*Plantago lanceolata* L. (020-084)	Whole plant	Plantaginaceae
*Artemisia annua* L. (008–007)	Leaf	Amaryllidaceae	*Cymbopogon tortilis* J. Presl (010–002)	Whole plant	Poaceae
*Nerium indicum* L. (018–097)	Leaf	Apocynaceae	*Delphinium maackianum* Regel (012–093)	Whole plant	Ranunculaceae
*Chrysanthemum boreale* Makino (004–039)	Whole plant	Asteraceae	*Hovenia dulcis* Thunberg (015–094)	Stem-bark	Rhamnaceae
*Chrysanthemum coronarium* L. (034–061)	Whole plant	Asteraceae	*Citrus unshiu* Marc (018-017)	Leaf-stem	Rutaceae
*Chrysanthemum indicum* L. (011–005)	Whole plant	Asteraceae	*Zanthoxylum piperitum* (L.) De Candolle (011–088)	Leaf	Rutaceae
*Chrysanthemum morifolium* Ramat (032–009)	Whole plant	Asteraceae	*Sapindus mukorossi* Gaertner (021–040)	Leaf-stem	Sapindaceae
*Tagetes erecta* L. (035-092)	Whole plant	Asteraceae	*Capsicum annum* L. (026-010)	Leaf-stem	Solanaceae
*Humulus japonicus* Siebold & Zucc. (008–095)	Leaf-stem	Cannabaceae	*Datura metel* L. (037-098)	Aerial part	Solanaceae
*Cleome spinosa* Jacquin (033-098)	Aerial part	Cleomaceae	*Nicotiana tabacum* L. (036–022)	Leaf-stem	Solanaceae
*Citrullus vulgaris* Schrader (035–064)	Whole plant	Cucurbitaceae	*Alnus japonica* Thunberg (003–084)	Leaf	Betulaceae
*Momordica charantia* L. (034–065)	Whole plant	Cucurbitaceae	*Arisaema takeshimense* Nakai (001–136)	Leaf	Araceae
*Rhododendron micranthum* Turcz (003–023)	Leaf-stem	Ericaceae	*Xylosma congestum* (Lour.) Merrill (001–113)	Leaf	Flacourtiaceae
*Ricinus communis* L. (018–093)	Leaf	Euphorbeaceae	*Acer takeshimense* Nakai (001–128)	Leaf	Aceraceae

### Laboratory bioassay

#### Evaluation of single plant extracts

Commercially produced plant extracts were diluted in our laboratory using methanol (99.5%, Daejung Chemicals and Metals Co. Ltd., Siheung, Republic of Korea) to make a 2 mg/ml stock solution. First instar (< 5 h old) larvae and adult male or female moths (3–5 d old) of *G*. *molesta* were used in our bioassays. Sex of adults used in bioassays was determined at the pupal stage by confirming the presence of an additional posterior abdominal segment in males [20]. Bioassays consisted of exposure of target life stage to an extract in scintillation glass vials (20 ml), to which 100 μl of each plant extract’s stock solution has been applied and allowed to air-dry, with rotation, for 2.5 h before the assay. This process allowed the methanol to fully evaporate, leaving the plant extract as a residue on the inner surface of the vial, after which five first instar (< 5 h old) larvae or adults were place in each vial. The vials were kept in the desiccators at 25.3 ± 0.03°C and 70.2 ± 0.8% RH for larvae and 25.2 ± 0.02°C and 70.5 ± 0.9% RH for adults in the incubator. Methanol was used as a negative control and the synthetic insecticide λ-cyhalothrin as a positive control. Mortality was observed every 4 and 24 h for larvae and adult, respectively, until death of all insects in the negative control. Bioassays were conducted with 30 larvae and 30 adults per treatment with six replications (5 insects/ replication).

#### Tests with mixed extracts

The synergistic effects of mixtures of pairs of plant extracts were determined by the co-toxicity coefficient (CTC) method in the laboratory [21, 22]. The mixture of two plant extracts, at a 1:1 ratio and concentration of 2 mg/ml, was applied to larvae and adults of *G*. *molesta*. Bioassays were conducted in glass scintillation vials similar to those described in the previous section.

Calculation of co-toxicity coefficients Sun and Johnson [21].

We calculated the co-toxicity coefficients of extract mixtures as per Sun and Johnson [21]: Co-toxicity coefficient (CTC) = (LT_50_ of toxicant alone / LT_50_ of toxicant in the mixture) × 100 (CTC = 100, similar action; CTC >100, synergistic action; CTC<100, antagonism).

### Greenhouse bioassay

Plant extracts were also evaluated in greenhouse trials. Before the experiment, transparent film (O.H.P film, 210 mm × 297 mm, PP2910, 3M, Seoul, Republic of Korea) was put inside the acrylic cage used for adult moths as an oviposition substrate. Eggs of this film were then collected and used for experiments. After spraying 4 ml of a given plant extract (at a concentration of 2 mg/ml) on each twig of a potted peach tree, 25 eggs were attached to five twigs (5 eggs/twig) for each treatment. Tangle trap (Tanglefoot Company, Grand Rapids, Michigan, USA) was applied at the bottom of the twig to prevent hatched larva from escaping. After 7 d, twig infestation rates were determined.

### Assessment of oviposition deterrence in laboratory assay

Oviposition deterrence effects of plant extracts were evaluated in the laboratory. Tests were carried out using peach tree twigs with five leaves each. At first, twigs (length of 10–12 cm) were put in conical flask (250 ml) filled with water to keep the twigs alive for about 7 d. Then, 4 ml of plant extracts were sprayed at a concentration of 2 mg/ml on the twigs, after which twigs were kept for 2.5 h to allow the plant extract to dry or 5 h to allow the positive control of λ-cyhalothrin to dry. Twigs in the conical flask were then placed on plastic trays and covered with ventilated acrylic cylinder cages (25.5 H × 8.5 D cm). Five mated female moths that had begun to lay eggs the previous day, together with five males, were released into each acrylic cylinder cage and held at 25.4 ± 0.1°C, 42.1 ± 0.4% RH, and a 16:8 h (L:D) photoperiod in the growth chamber. We then observed the number of eggs laid on each twig or on the wall of a cage every 24 h for up to five days. The experiments were replicated two times.

### Assessment of oviposition deterrence in a greenhouse assay

The oviposition deterrence of plant extracts was also evaluated under greenhouse conditions. Four ml of each plant extract were sprayed onto potted peach plants at a concentration of 2 mg/ml and plants were then allowed to dry for 2.5 h. After fully drying, plants were covered with a pipe framed cage (47.0 L × 47.0 W × 115.0 L cm) screened with white-colored nylon fabric Then five female moths (mated and started oviposition one day before) and five males were released inside the cage. We then observed the number of eggs laid on each twig or on the wall of a cage every 24 h for up to five days. The experiments were replicated two times.

### HPLC analysis

#### Instrumentation

An Agillent 1200 series (Agilent, Santa Clara, CA) HPLC system was equipped with bin pump (G1312A), degasser (G13796), column oven (250 × 4.6 mm and 5 μm particle size, Agilent, Santa Clara, CA), and diode array detector (G1315B). Agilent ChemStation software was used for data acquisition and system suitability calculations.

#### Chromatographic parameters

Reverse phase high performance liquid chromatography (RP-HPLC) was used for the analysis for *N*. *tabacum* and *A*. *sativum* extract according to the method described by Tanbwekar et al. [23] with a minor modification. In our study, column temperature was used at 25°C instead of 35°C. Column was used with flow rate of 1 ml/minute. Diode array detector in range of 200–800 nm was used for determining peak purity. Injection volume was 20 μl where phosphate buffer (pH 6.8; 10nm) with methanol (35.65% v/v) was used as mobile phase.

### Statistical analysis

Larval mortality data were corrected using Abbott’s formula [24] and then were used to calculate the lethal median time (LT_50_) using SAS 9.4 software [25]. Infestation of twigs in greenhouse and number of eggs laid on substrates in the oviposition deterrence experiment in the laboratory were analyzed using a Chi-square test with a post-hoc multiple comparison test analogous to Tukey’s test [26].

In the oviposition deterrence experiment in the greenhouse, the number of eggs was analyzed using single factor analysis of variance (ANOVA) and differences in the mean number of eggs were determined by Tukey’s test using Proc MIXED of SAS 9.4 [25]. Before analysis, normality and homogeneity were tested using a Kolmogorov-Smirnov test (*P* = 0.150) and a Levene test (*P* = 0.442).

## Results

### Laboratory bioassay

#### Evaluation of single plant extracts

**Fig 1:**
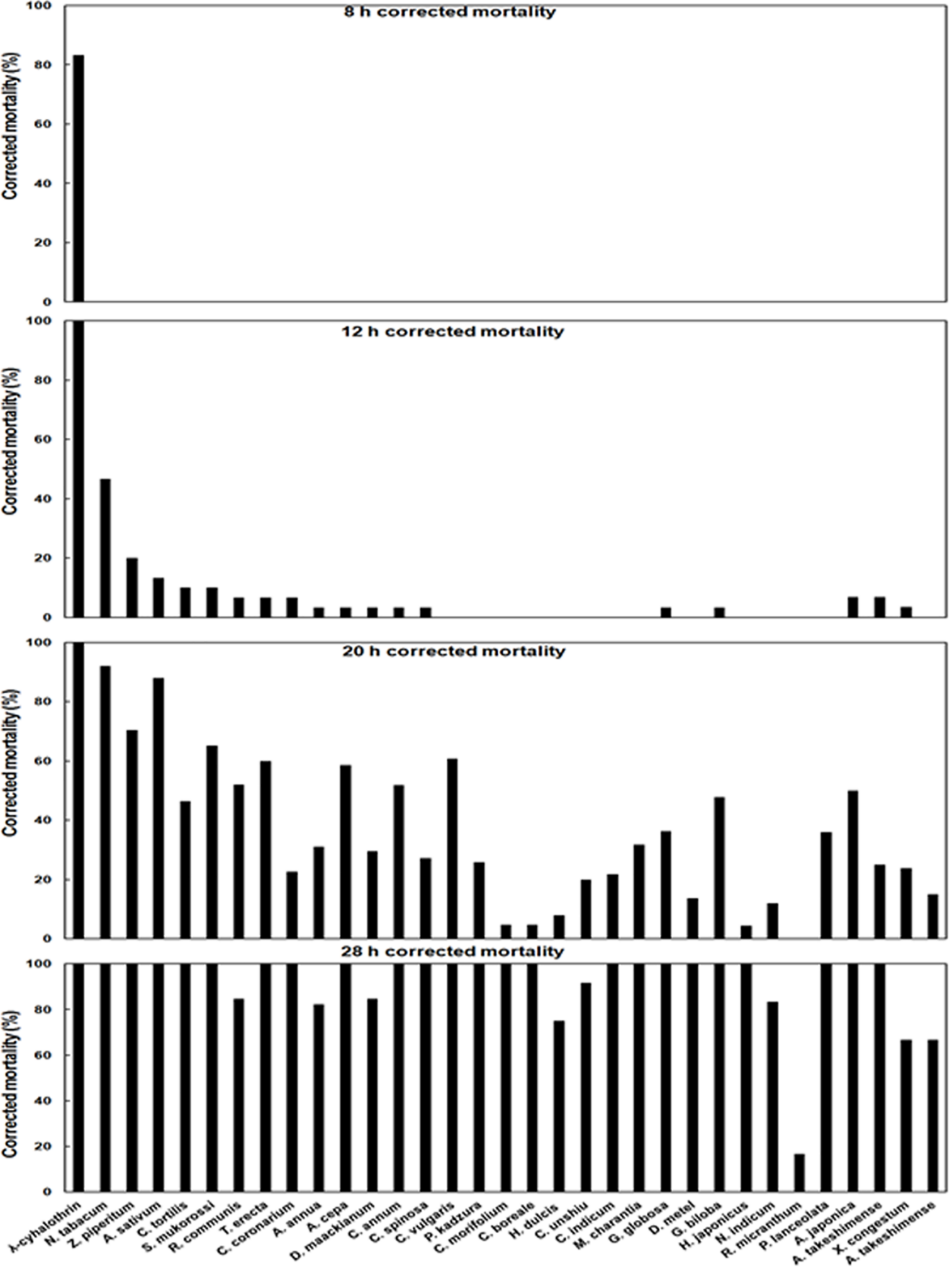
Efficacy of different plant extracts against *Grapholita molesta* 1st instar larvae over time.

Among the 32 plant extracts tested, *Nicotiana tabacum* L., *Allium sativum* L., and *Zanthoxylum piperitum* (L.) De Candolle showed the highest mortality on first instar larva (Table 3). The LT_50_ values of *N*. *tabacum*, *A*. *sativum*, and *Z*. *piperitum* were 12.9 h (*χ^2^* = 9.99, *df* = 4, *P* = 0.041), 15.6 h (*χ^2^* = 4.02, *df* = 4, *P* = 0.403), and 16.1 h (*χ^2^* = 17.02, *df* = 4, *P* = 0.002), respectively. The LT_50_ value of *Sapindus mukorossi* Gaertner was 17.5 h (*χ^2^* = 10.04, *df* = 5, *P* = 0.074), which was significantly higher than *N*. *tabacum* or *A*. *sativum*. *Nicotiana tabacum* showed highest corrected mortality of 92.0% followed by *A*. *sativum* (88.0%), *Z*. *piperitum* (70.4%), and *S*. *mukorossi* (65.2%) within 20 h (Fig 1). For the positive control, λ-cyhalothrin, 100% corrected mortality was found within 12 hours. On the basis of the LT_50_ value, *N*. *tabacum*, *A*. *sativum*, *Z*. *piperitum*, and *S*. *mukorossi* were chosen as the four most effective plant extracts against first instar larvae of *G*. *molesta*, and these extracts were further evaluated in subsequent experiments.

**Table 3:** Statistical comparison of methanolic plant extracts (200μg/vial) against the 1st instar larva of *Grapholita molesta* by scintillation glass vial assay.

Treatment	LT_50_	95% C.I	Slope ± SE	*χ^2^* (df)
λ-cyhalothrin	5.32a	4.92–5.72	6.21 ± 0.58	2.35
*Nicotiana tabacum*	12.92b	11.57–14.14	9.07 ± 1.09	9.99 (4)
*Allium sativum*	15.57c	15.03–16.09	11.16 ± 0.88	4.02 (4)
*Zanthoxylum piperitum*	16.09bcd	14.07–18.15	8.57 ± 1.40	17.02 (4)
*Sapindus mukorossi*	17.48d	16.32–18.62	9.74 ± 0.98	10.04 (5)
*Tagetes erecta*	17.95de	17.29–18.59	8.91 ± 0.64	8.24 (5)
*Allium cepa*	18.52de	17.94–19.09	11.30 ± 0.83	5.51 (5)
*Citrullus vulgaris*	18.70de	18.12–19.26	14.91 ± 1.15	6.52 (5)
*Cymbopogon tortilis*	19.07de	17.08–21.21	7.94 ± 1.19	20.49 (5)
*Capsicum annum*	19.09de	18.49–19.69	10.87 ± 0.80	8.16 (5)
*Alnus japonica*	19.09de	17.53–20.71	8.73 ± 1.09	14.41 (5)
*Ricinus communis*	19.36de	18.61–20.09	7.50 ± 0.50	8.66 (6)
*Gomphrena globosa*	19.50de	17.61–21.47	10.14 ± 1.61	23.04 (5)
*Ginkgo biloba*	19.78de	18.19–21.37	11.59 ± 1.65	18.63 (5)
*Momordica charantia*	20.55e	18.86–22.31	11.76 ± 1.84	20.45 (5)
*Plantago lanceolata*	20.90e	20.36–21.44	14.56 ± 1.15	6.25 (5)
*Piper Kadzura*	21.35e	19.87–22.91	13.38 ± 1.98	17.72 (5)
*Cleome spinosa*	21.50de	16.50–35.96	12.04 ± 4.16	103.07 (5)
*Arisaema takeshimense*	21.51de	17.56–27.97	9.16 ± 2.52	64.14 (5)
*Delphinium maackianum*	21.69e	20.15–23.28	9.16 ± 1.07	17.54 (6)
*Chrysanthemum indicum*	21.87e	19.05–25.61	10.72 ± 2.52	42.42 (5)
*Chrysanthemum coronarium*	22.25de	17.38–34.55	8.92 ± 2.84	80.31 (5)
*Artemisia annua*	22.67e	20.31–25.25	9.42 ± 1.64	37.51 (6)
*Datura metel*	22.77e	20.29–25.93	13.98 ± 3.27	41.16 (5)
*Citrus unshiu*	22.86e	21.39–24.36	12.84 ± 1.72	21.27 (6)
*Xylosma congestum*	23.09e	20.87–25.61	8.68 ± 1.39	30.74 (6)
*Chrysanthemum boreale*	23.17e	16.79–32.93	18.71 ± 6.76	94.56 (5)
*Hovenia dulcis*	24.02e	22.09–26.08	12.30 ± 2.03	31.59 (6)
*Nerium indicum*	24.15e	23.61–24.69	16.47 ± 1.28	4.80 (6)
*Humulus japonicus*	24.48e	22.91–26.28	27.58 ± 6.45	29.28 (5)
*Acer takeshimense*	25.02e	23.65–26.45	13.93 ± 1.88	18.30 (6)
*Rhododendron micranthum*^a^	-	-	-	-
*Chrysanthemum morifolium*^a^	-	-	-	-

LT_50_ values followed by different lower case letters are significantly different among treatments

^a^Larvae died faster than control, so LT_50_ was not calculated

**Fig 2:**
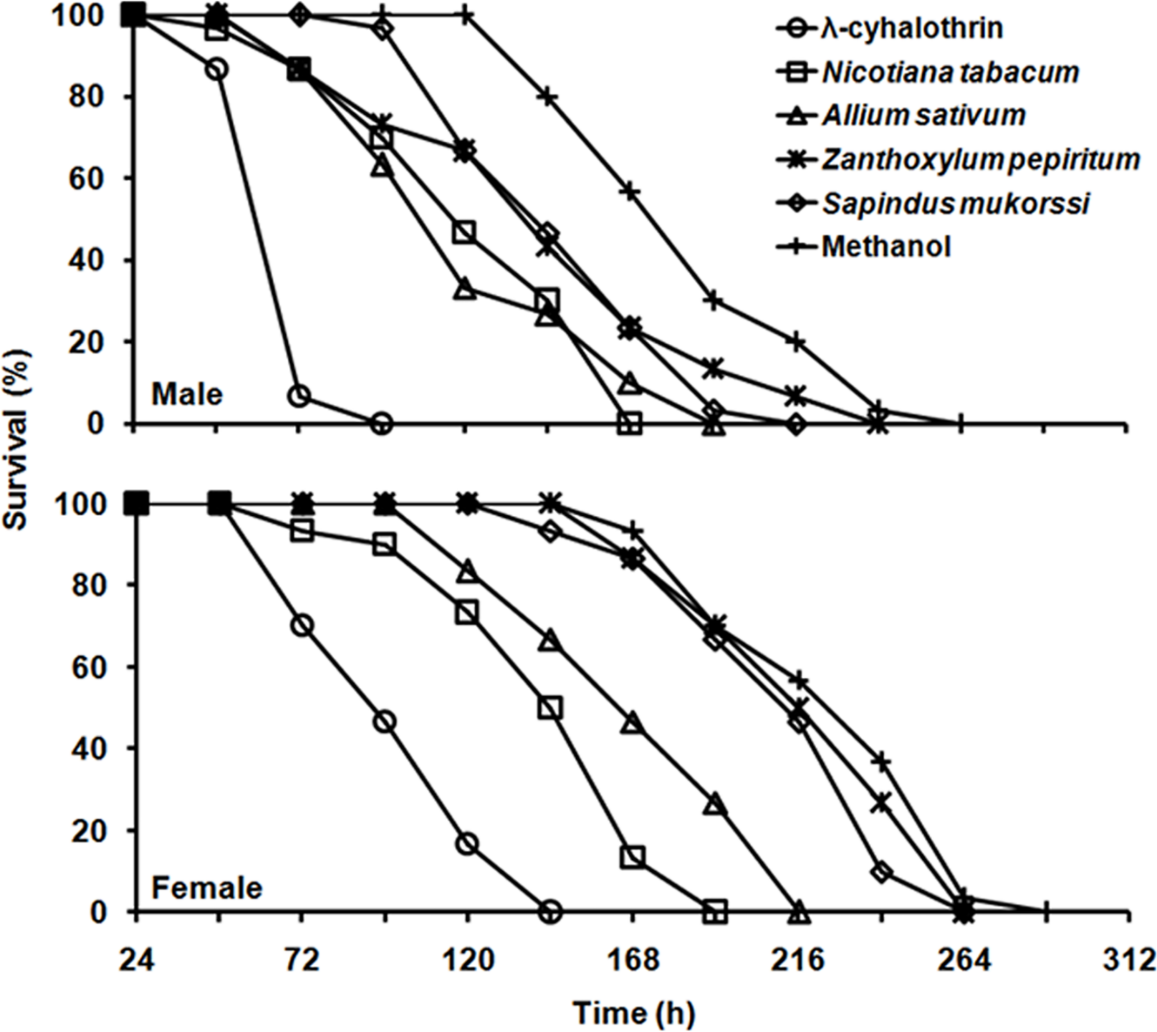
Survivorship of adult male and female of *Grapholita molesta* after exposure to single applications of plant extracts.

In the adult assay, 100 and 96.7% of adult males survived 24 and 48 h, respectively, but only 30.0% of adult males survived 144 h when held in vials treated with *N*. *tabacum* (Fig 2). *Allium sativum* and *N*. *tabacum* both caused higher mortality than *S*. *mukorossi* and methanol on adult males with LT_50_ values of 107.5 (*χ^2^* = 3.08, *df* = 6, *P* = 0.799) and 109.9 h (*χ^2^* = 7.46, *df* = 5, *P* = 0.189), respectively (Table 4). In case of adult females, *N*. *tabacum* and *A*. *sativum* were also significantly more effective than other plant extracts, with LT_50_ values of 131.9 (*χ^2^* = 14.39, *df* = 6, *P* = 0.026) and 158.3 h (*χ^2^* = 5.96, *df* = 7, *P* = 0.544), respectively (Table 4). Irrespective of treatments, adult male *G*. *molesta* adult died faster than females (Fig 2).

**Table 4:** Statistical comparison of tested methanolic plant extracts against adult *Grapholita molesta*.

Tested on	Treatment	LT_50_	95% C.I.	Slope ± SE	*χ^2^* (df)
Male	λ-cyhalothrin	57.01a	53.11–61.29	14.90 ± 2.53	0.01 (2)
	*Allium sativum*	107.49b	99.15–115.55	7.03 ± 0.83	3.08 (6)
	*Nicotiana tabacum*	109.96bc	101.17–119.41	6.43 ± 0.85	7.46 (5)
	*Zanthoxylum piperitum*	126.35cd	116.72–135.85	6.05 ± 0.63	5.85 (8)
	*Sapindus mukorossi*	137.66de	130.26–144.81	10.99 ± 1.33	2.96 (7)
	Methanol	174.73f	166.86–182.33	12.28 ± 1.39	3.17 (9)
Female	λ-cyhalothrin	88.80a	81.91–95.44	8.53 ± 1.20	3.77 (4)
	*Nicotiana tabacum*	131.93b	115.23–150.72	8.63 ± 1.65	14.39 (6)
	*Allium sativum*	158.34bc	150.23–166.77	10.44 ± 1.33	5.96 (7)
	*Sapindus mukorossi*	201.46d	193.66–209.54	13.87 ± 1.65	7.67 (9)
	*Zanthoxylum piperitum*	209.58de	201.74–217.78	14.49 ± 1.83	5.33 (9)
	Methanol	215.49ef	207.77–223.23	15.15 ± 1.76	6.66 (10)

LT_50_ values followed by different letters are significantly different among treatment.

#### Evaluation of mixed extracts

**Fig 3:**
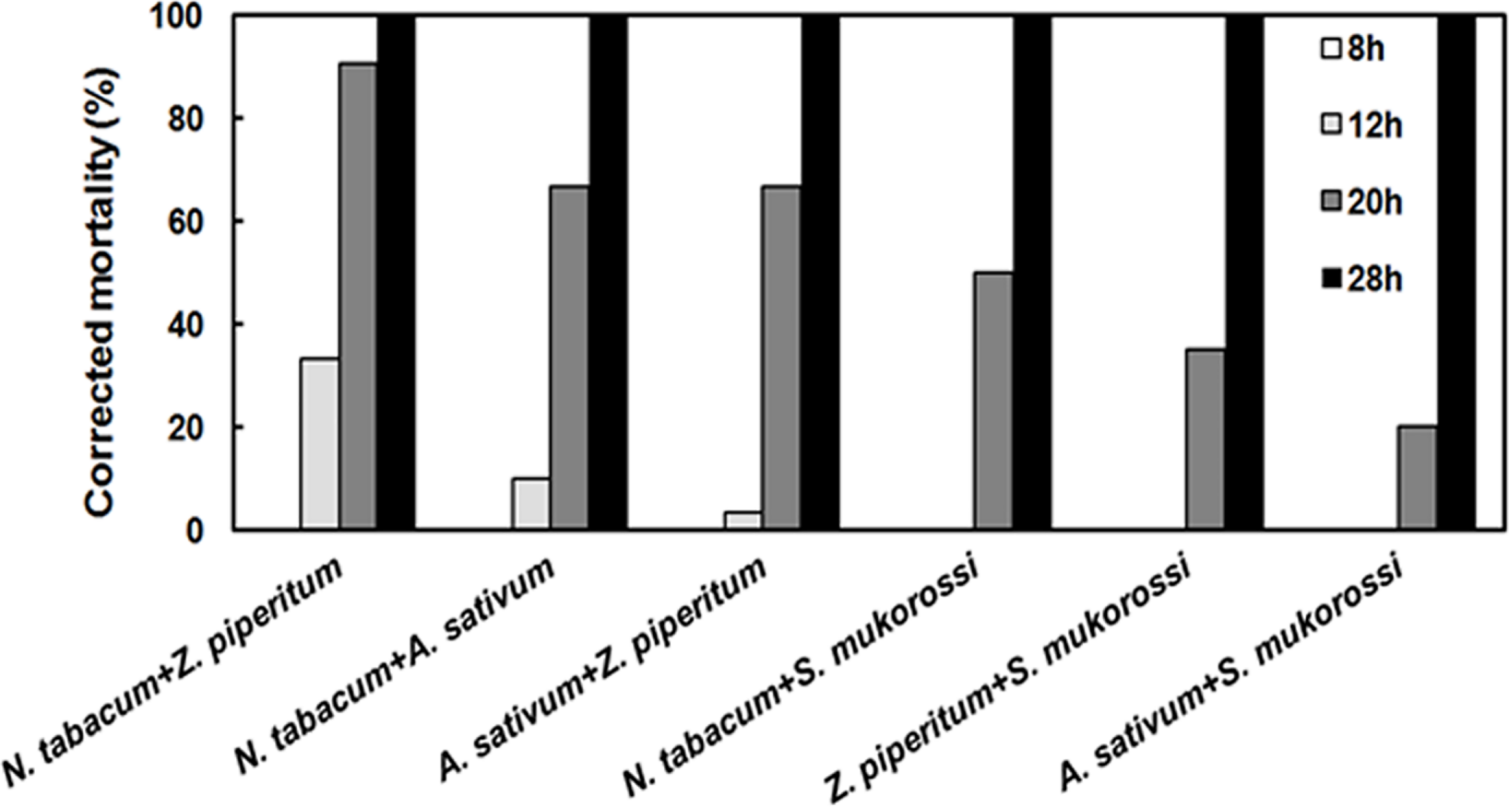
Corrected mortality (%) of combinations of plant extracts against first instar larvae of *Grapholita molesta*.

**Fig 4:**
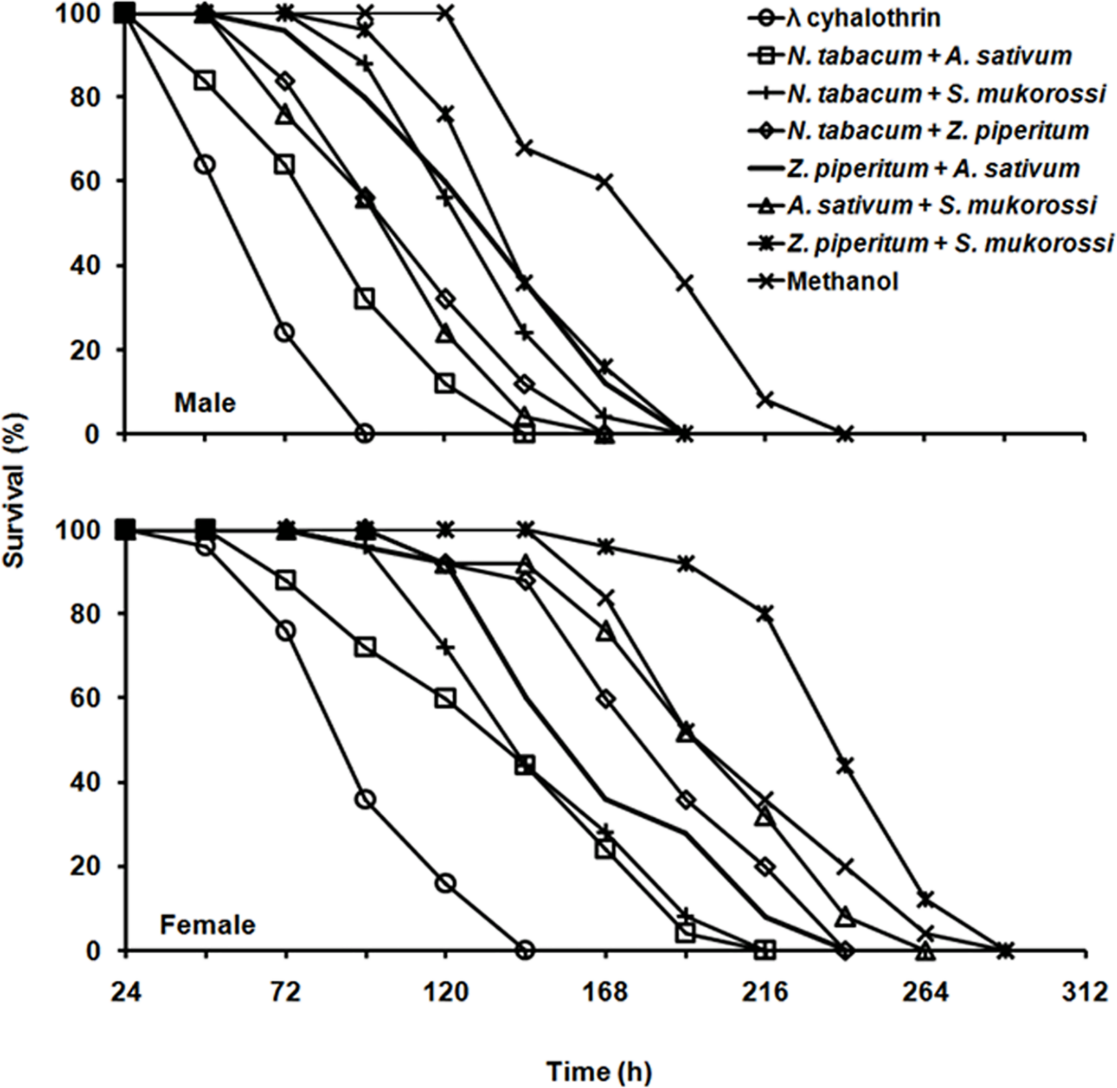
Survivorship of adult male and female of *Grapholita molesta* on mixed application of plant extracts.

We also evaluated the effect of mixtures of plant extracts on first instar larvae (< 5 h old) and on both male and female adults (< 5 d old) of *G*. *molesta*. The first instar larvae of *G*. *molesta* died faster when treated with the mixture of *N*. *tabacum+Z*. *piperitum*, with corrected mortality of 90.5% at 20 h after treatment (Fig 3). The LT_50_ value of the mixture of *N*. *tabacum+Z*. *piperitum* was 14.3 h (*χ^2^* = 11.32, *df* = 4, *P* = 0.023), but the co-toxicity coefficient value was 90.5 indicating that there was no synergistic effect of the mixture of *N*. *tabacum*+*Z*. *piperitum*. The lethal median time (LT_50_) was 76.7 h (*χ^2^* = 2.87, *df* = 4, *P* = 0.579) for adult males, significantly different from the mixture of *N*. *tabacum*+*A*. *sativum* (Table 5) in which all adults died within 144 h (Fig 4). The co-toxicity coefficient value of *N*. *tabacum*+*A*. *sativum* was 140.1, indicating a synergistic effect of the mixture of these two extracts. However, in case of adult females, the LT_50_ value was not significantly different between the mixture of *N*. *tabacum*+*A*. *sativum* and the mixture of *A*. *sativum*+*S*. *mukorossi* (Table 5). The mixture of *N*. *tabacum*+*A*. *sativum* showed 100% mortality within 144 h (Fig 4). The co-toxicity coefficient value of *N*. *tabacum*+*A*. *sativum* mixture was 107.5, indicating a synergistic effect of the mixture (Table 5), but, from the C. I. value, the mixture of *N*. *tabacum*+*A*. *sativum* was not significantly different from the single extract of *N*. *tabacum*. Here, we also found that adult males died faster than adult females in mixed extract treatment. From the above results, the mixture of *N*. *tabacum*+*A*. *sativum* would be the best choice for use against adult males, but the mixture of *N*. *tabacum*+*A*. *sativum* and *N*. *tabacum* by itself were both equally lethal to adult females.

**Table 5:** Statistical comparison of tested methanolic plant extracts (mixture) against *Grapholita molesta*.

Tested on	Treatment	LT_50_	95% C.I.	Slope ± SE	χ^2^ (df)	Co-toxicity coefficient
Larva^a^, first instar	λ-cyhalothrin	5.32a	4.92–5.72	6.21 ± 0.58	2.35	-
	*N*. *tabacum+Z*. *piperitum*	14.27b	12.78–15.65	9.03 ± 1.17	11.32(4)	90.54
	*N*. *tabacum+A*. *sativum*	18.20c	16.52–19.90	8.31 ± 1.08	16.26(5)	70.99
	*A*. *sativum+Z*. *piperitum*	18.04c	17.47–18.60	11.40 ± 0.84	2.51(5)	86.31
	*N*. *tabacum+S*. *mukorossi*	18.99cd	17.83–20.10	12.44 ± 1.44	11.38(5)	68.04
	*A*. *sativum+S*. *mukorossi*	21.80cde	19.81–24.05	12.95 ± 2.45	28.37(5)	71.42
	*Z*. *piperitum+S*. *mukorossi*	21.65cdef	18.56–25.79	9.65 ± 2.34	43.92(5)	74.32
Adult, male	λ-cyhalothrin	54.87a	48.10–60.78	7.97 ± 1.54	1.67 (2)	-
	*N*. *tabacum*+*A*. *sativum*	76.70b	68.37–84.76	6.19 ± 0.91	2.87 (4)	140.14
	*A*. *sativum*+*S*. *mukorossi*	94.48c	86.63–101.98	8.35 ± 1.17	3.04 (5)	113.77
	*N*. *tabacum*+*Z*. *piperitum*	100.13cd	91.88–108.11	8.06 ± 1.14	1.84 (5)	109.82
	*N*. *tabacum*+*S*. *mukorossi*	122.87e	115.50–129.94	12.15 ± 1.72	0.74 (6)	89.49
	*Z*. *piperitum*+*A*. *sativum*	123.65e	114.92–132.41	8.58 ± 1.15	2.86 (6)	86.93
	*Z*. *piperitum*+*S*. *mukorossi*	135.43ef	127.90–142.82	12.69 ± 1.82	1.28 (6)	93.30
	Methanol	170.30g	161.87–178.65	12.45 ± 1.66	6.74 (8)	-
Adult, female	λ-cyhalothrin	86.03a	78.37–93.50	8.01 ± 1.18	1.79 (4)	-
	*N*. *tabacum*+*A*. *sativum*	122.69b	112.66–132.71	6.51 ± 0.80	6.51 (7)	107.53
	*A*. *sativum*+*S*. *mukorossi*	140.15bc	131.65–148.40	10.36 ± 1.36	1.71 (7)	112.98
	*Z*. *piperitum*+*A*. *sativum*	156.65cd	147.49–165.68	9.85 ± 1.21	3.55 (8)	101.08
	*N*. *tabacum*+*Z*. *piperitum*	175.50e	166.48–184.81	11.21 ± 1.46	4.49 (8)	75.17
	*N*. *tabacum*+*S*. *mukorossi*	187.83ef	178.40–197.59	10.81 ± 1.31	9.03 (9)	70.24
	*Z*. *piperitum*+*S*. *mukorossi*	231.07h	223.06–239.38	18.18 ± 2.40	7.66 (10)	90.70
	Methanol	200.61fg	191.94–209.00	13.99 ± 1.72	2.74 (10)	-

LT_50_ values followed by different letters are significantly different among treatment

^a^The LT_50_ value was calculated using corrected mortality

### Greenhouse bioassay

In the greenhouse bioassay, infestation levels of twigs were significantly reduced when twigs were sprayed with either *N*. *tabacum* or *A*. *sativum* (*χ^2^* = 30.74, *df* = 5, *P* < 0.001) compared to the negative control (Table 6). However, we found no significant differences among the plant extracts (*χ^2^* = 7.19, *df* = 3, *P* = 0.066).

**Table 6:** Efficacy evaluation of plant extracts on infestation rate of peach twigs in greenhouse.

Treatment	Hatchability (%)	Infestation rate
λ-cyhalothrin	88.0	0.09 (2/22)d
*Nicotiana tabacum*	88.0	0.27 (6/22)cd
*Allium Sativum*	84.0	0.38 (8/21)bdc
*Zanthoxylum piperitum*	88.0	0.45 (10/22)abcd
*Sapindus mukorssi*	84.0	0.67 (14/21)abc
Control	88.0	0.82 (18/22)a

Means within a column with different letters differ significantly (*P* < 0.05)

### Oviposition deterrence in the laboratory

**Fig 5:**
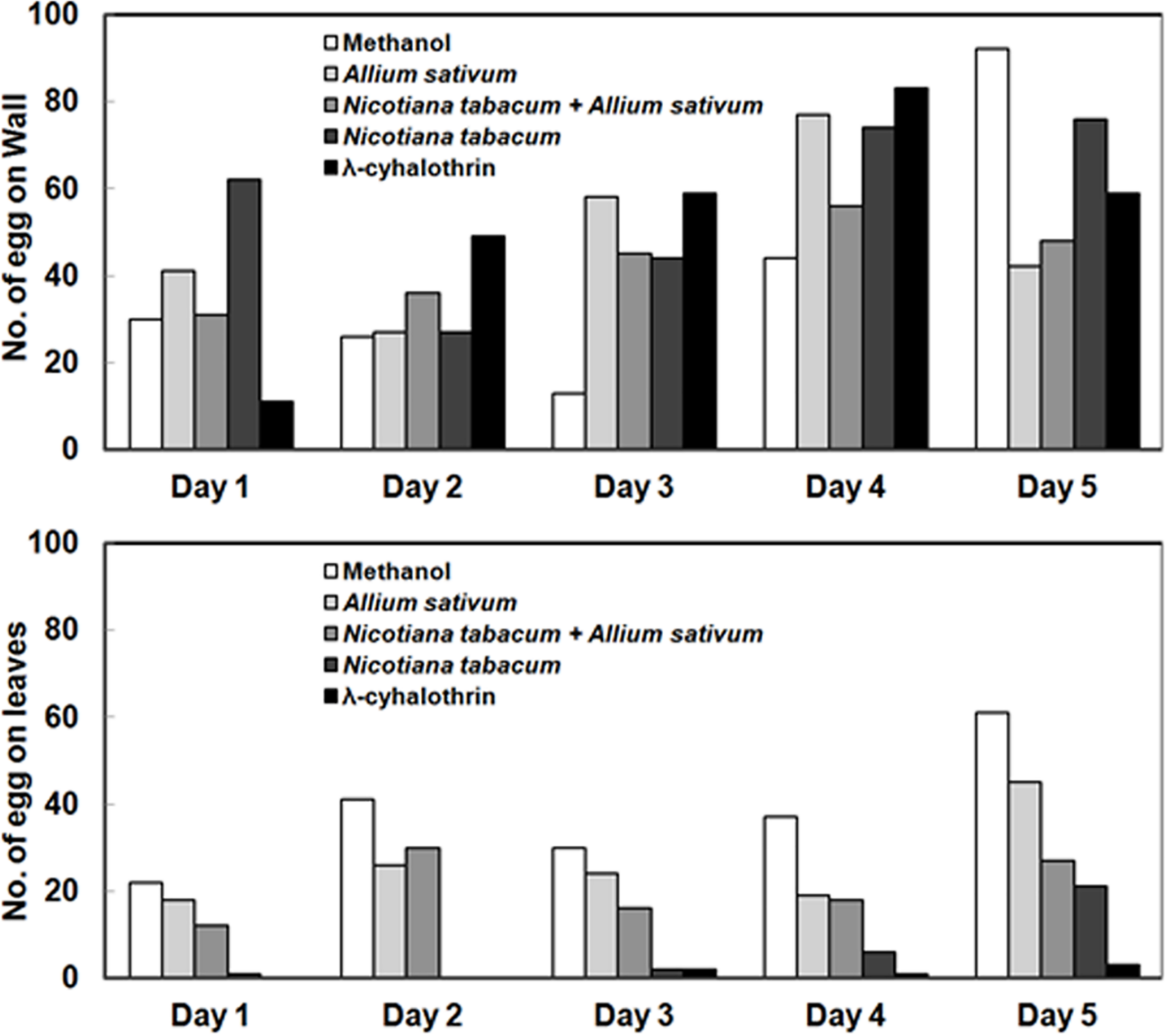
Daily egg laying on cage walls and leaves up to five days.

From the above experiments we found that *N*. *tabacum*, *A*. *sativum*, and the mixture of *N*. *tabacum*+*A*. *sativum* provided the best control of adult *G*. *molesta*, so, these treatments were compared in an oviposition deterrence test in the laboratory. Mated females laid only 29 eggs on the leaves treated with *N*. *tabacum*, significantly fewer than all other plant extracts, and an 85% reduction compared to the methanol control (*χ^2^* = 236.50, *df* = 4, *P* < 0.001) (Table 7). We found *N*. *tabacum* to be very effective in reducing oviposition, at levels similar to those provided by λ-cyhalothrin, for up to three days (Fig 5).

**Table 7:** Deterrent effect of plant extract on oviposition of *G*. *molesta* in laboratory.

Treatment	Total no. of eggs produced	% eggs on wall	% eggs on leaves
λ-cyhalothrin	267	97.75a	2.25a
*Nicotiana tabacum*	312	90.71b	9.29b
*N*. *tabacum*+*A*. *sativum*	319	67.71c	32.29c
*Allium sativum*	377	64.99c	35.01c
methanol	396	51.77d	48.23d

Means within a column with different letters differ significantly (*P* < 0.05)

### Oviposition deterrence the greenhouse

The number of eggs laid by adult mated females was significantly lower for all plant extracts compared to the negative control (*F* = 9.82, *df* = 4, 9, *P* = 0.014), and the percentage of leaves with eggs and the total number of eggs laid were reduced in the *N*. *tabacum* treatment by 71 and 90%, respectively, compared to the methanol control (Table 8).

**Table 8:** Deterrent effect of plant extract on oviposition of *G*. *molesta* on greenhouse.

Treatment	No. of leaves/twig	Percent of twigs of which leaves with egg	Percent of leaves with egg	Total no. of eggs reproduced
λ-cyhalothtrin	9.36 (103/11)	0.00 (0/11)a	0.00 (0/103)a	0c
*Nicotiana tabacum*	8.56 (94/11)	36.36 (4/11)ab	8.51 (8/94)b	18b
*Allium sativum*	6.79 (95/14)	57.14 (8/14)b	15.79 (15/95)b	28b
*N*. *tabacum*+*A*. *sativum*	9.00 (117/13)	69.23 (9/13)b	19.67 (23/117)bc	42b
methanol	7.15 (93/13)	46.15 (6/13)b	29.03 (27/93)c	184a

Means within a column with different letters differ significantly (*P* < 0.05)

### HPLC analysis

**Fig 6:**
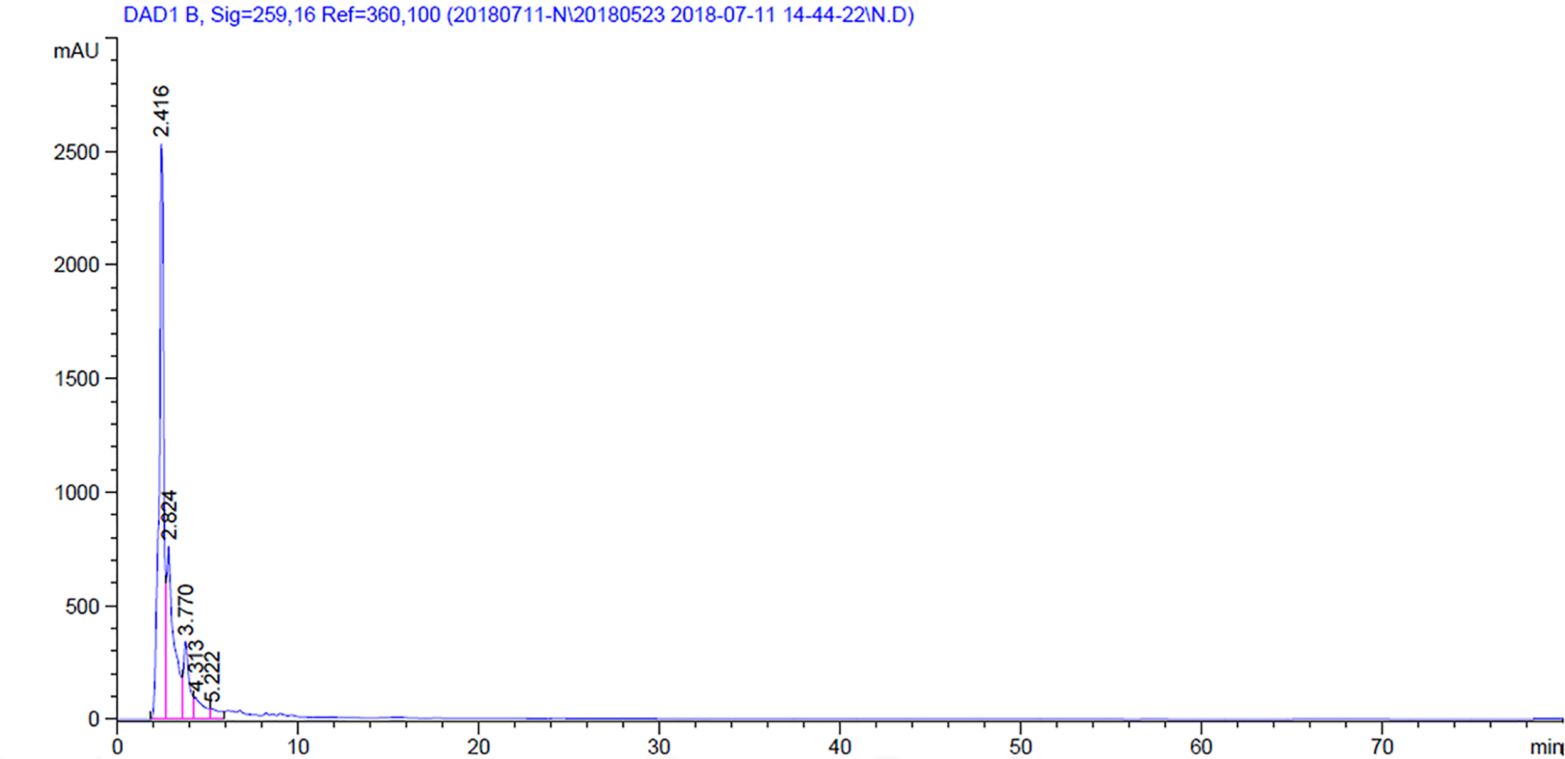
HPLC of methanol extract of *Nicotiana tabacum*.

**Fig 7:**
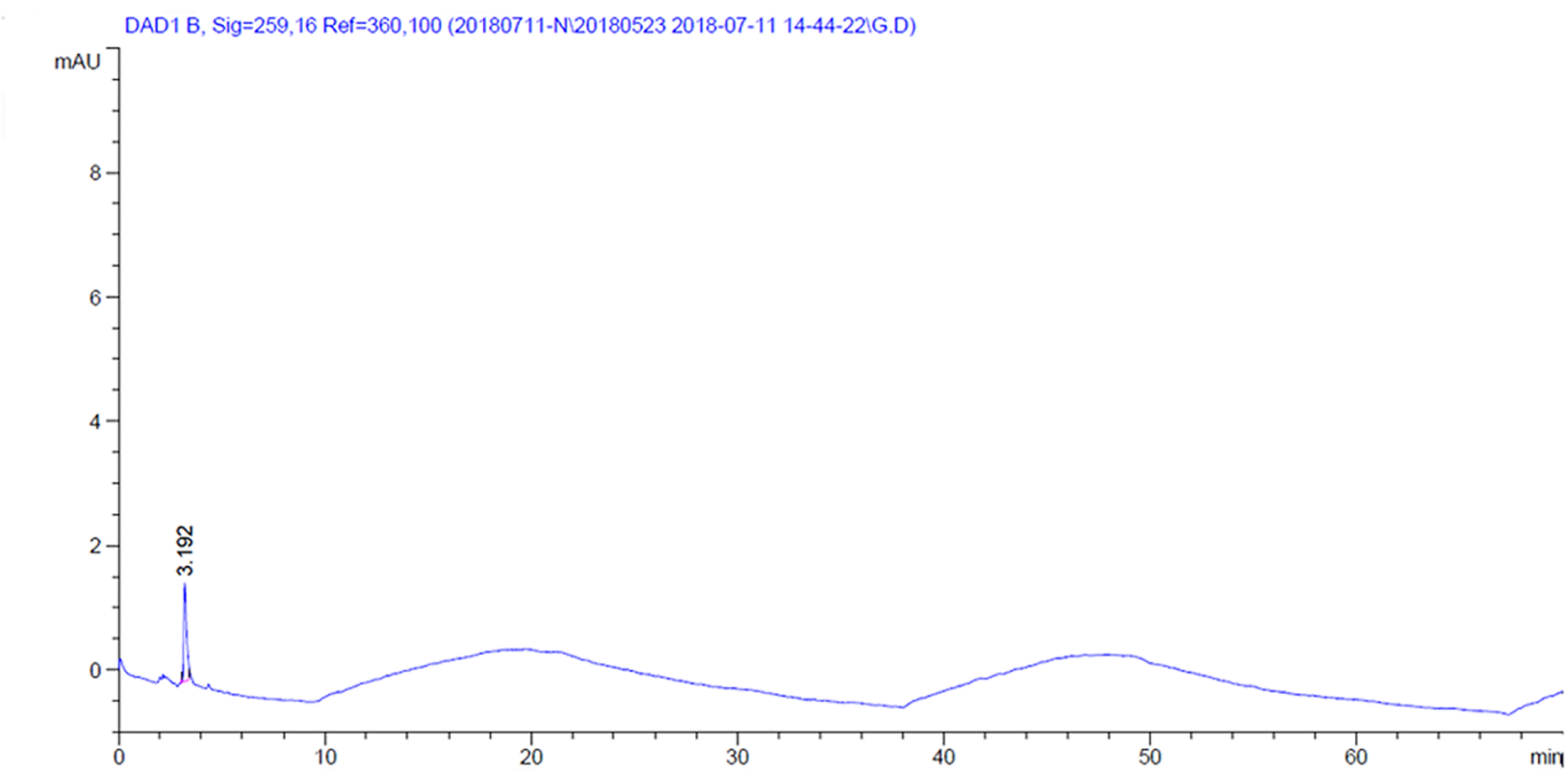
HPLC of methanol extract of *Allium sativum*.

Nicotine the major compound of *N*. *tabacum* appeared 56.3% at RT 2.42 min with two unidentified minor compounds at RT 2.83 min (27.01%) and 3.77 min (10.13%) (Fig 6). From *A*. *sativum*, the major compound allicin appeared 100% at RT 3.19 min (Fig 7).

## Discussion

The synthetic pesticide λ-cyhalothrin was more toxic than any of plant extracts to first instar larvae. Based on the comparison of plant extract LT_50_ values to that of λ-cyhalothrin, we selected *N*. *tabacum*, *A*. *sativum*, *Z*. *piperitum*, and *S*. *mukorossi* as the most effective botanical extracts for control of first instar larvae of *G*. *molesta*. Although the highest mortality was observed in larval stage of *G*. *molesta* from *N*. *tabacum* treatment, for both adult males and females *N*. *tabacum* and *A*. *sativum* were equally effective in a subsequent assay. *Nicotiana tabacum* has several modes of action. It can be a nerve poison [27, 28], stomach poison, or repellent [29]. Baskaran and Narayanasamy [29] found *N*. *tabacum* to be effective against aphids, thrips, psyllids, tingids, beetles, sawflies, and lepidopterans. Evaluation of *N*. *tabacum* against *G*. *molesta* has been made here for the first time. In addition, *N*. *tabacum* is easy to apply in the field. Amoabeng et al. [16] ground *N*. *tabacum* leaves in tap water containing 0.1% Sunlight^®^ detergent solution and sieved them through fine linen for immediate application to a cabbage field. This preparation resulted in 93.0% reduction of *Plutella xylostella* larvae, while λ-cyhalothrin reduced the same population by only 51.0%. The best efficacy was recorded with the extract of *N*. *tabacum* against *Cydia molesta* Busch. (98.3%) and *Anarsia lineatella* Zell. (99.0%) [30]. Vandenborre et al. [27] found that a jasmonate-inducible lectin named NICTABA present in tobacco leaf is responsible for the larval mortality of lepidopteran insects. Nevertheless, a major active compound of *N*. *tabacum* was nicotine, which mimics acetylcholine and activates the nicotinic acetylcholine receptor causing an influx of sodium ions to flood the receptor [28]. Methanolic extracts of *A*. *sativum* have also caused mortality of 81.0% against *Spodoptera litura* [31]. A constituent of the *A*. *sativum* extract, alliin (derived from the amino acid cysteine) is converted by an enzyme to allicin, which is believed to act as an antifeedant, repellent, and insecticide [32].

We did not find any synergistic effects of *N*. *tabacum* and *Z*. *piperitum* on first instar larvae of *G*. *molesta*. However, the mixture of *N*. *tabacum*+*A*. *sativum* showed synergistic effects on adult males. The reason for this difference in the effectiveness of the mixture of *N*. *tabacum*+*A*. *sativum* between larvae and adults is unknown, but might be caused by differences in physiological structure. Similarly, Derbalah [33], who found that an extract of *Bauhinia purpurea* L. showed 83 and 80% mortality on adult and pupal stages of *Trogoderma granarium* Everts, respectively, but only 33.0% mortality on the larval stage. Interestingly, extracts of *Caesalpinia gilliesii* (Hook.) showed lower mortality on adult and pupal stages (43.0 and 43.0%, respectively), than on larvae (80%).

We found no synergistic effect of *N*. *tabacum* and *Z*. *piperitum* on the first instar larvae of *G*. *molesta*, and similarly Noosidum et al. [34] found no synergistic effect of the mixture of *Litsea salicifolia* Roxb. (0.1%) and *Melaleuca leucadendron* L. (0.3%) against adult females of *Aedes aegypti* (L.). However, the synergistic effects of mixtures of plant extracts have been reported in other studies. Alim et al. [35] found that a mixture of neem plus crown flower at a 1:1 ratio showed synergistic effects on *Aleurodicus dispersus* adults. Zibaee and Khorram [36] also found that essential oils of *Eucalyptus globulus* Labill. and *Rosmarinus officinalis* L. showed synergistic effects on *Blattella germanica* L.

*Nicotiana tabacum* extract was effective in deterring oviposition in both laboratory and greenhouse assays, which suggests it would be effective at reducing *G*. *molesta* populations in the field. Similarly, Amoabeng et al. [16] found that *N*. *tabacum* extract reduced 93.0% of a *Plutella xylostella* population in a cabbage field. In other work in Uganda, a crude extract of *N*. *tabacum* showed similar effectiveness to the synthetic insecticides against a bruchid beetle (*Callosobruchus* sp.) [37]. Nevertheless, plant extracts can be harmful to other beneficials: *N*. *tabacum* found to be harmful on *Coccinella magnifica* Redtenbacher and *Episyrphus balteatus* De Geer compared to tap water but less harmful than synthetic insecticides [16].

In conclusion, among the 32 tested plant extracts, *N*. *tabacum* extract showed highest toxicity against the first instar and adult of *G*. *molesta*, and oviposition was greatly reduced after the spray in both laboratory and greenhouse. Nevertheless, formulation should be improved as methanolic extracts in this study is not appropriate for organic farming. Based on these results, we are suggesting that the extract of *N*. *tabacum* can be a good botanical insecticide against *G*. *molesta*.

## Supporting information

S1 File Test of single plant extract on larva, test of single plant extract on adult, test of combination of extracts on larva, test of combination of extracts on adult, Greenhouse evaluation of plant extracts, Oviposition deterrency in laboratory, Oviposition deterrency in greenhouse. (XLSX)
